# In vitro starch digestion in sorghum flour from Algerian cultivars

**DOI:** 10.1002/fsn3.104

**Published:** 2014-03-17

**Authors:** Rachid Souilah, Djaffar Djabali, Badreddine Belhadi, Hind Mokrane, Nadia Boudries, Boubekeur Nadjemi

**Affiliations:** 1 Laboratoire de Recherche sur Les Produits Bioactifs et Valorisation de la Biomasse, Département de Chimie, Ecole Normale Supérieure de KoubaAlgiers, Algeria; 2 Laboratoire d'Etudes et Développement des Techniques d'Epuration et de Traitement des Eaux et Gestion Environnementale, Département de Chimie, Ecole Normale Supérieure de KoubaAlgiers, Algeria; 3 Laboratoire de Qualité et sécurité des Produits agroalimentaire, Unité d'Analyse, Qualité et Risques, Université de Liège-Gembloux Agro-Bio Tech. 02 Passage des déportésB-5030, Gembloux, Belgique

**Keywords:** First-order model, grain quality, kinetic parameters, sorghum, starch digestion

## Abstract

This work aims to evaluate starch digestion in whole sorghum grains. Nine sorghum cultivars were sampled from the Sahara of Algeria. The structural characteristics of sorghum grains were measured. Total starch (TS) varied between 67.67% and 74.82%, digestible starch (DS) between 64.34% and 69.70%, and resistant starch (RS) ranged from 2.55% to 7.98%. The kinetic of starch digestion displayed first-order model. For all sorghum cultivars, starch were digested with different extents, DS at infinite time (*D*_∞_) ranged from 52.58 to 102.13 g/100 g dry starch, while the hydrolysis index (HI) ranged from 41.55% to 76.93% and high average glycemic index (GI_avg_) ranged from 65.97 to 94.14. The results showed that there are differences in grain quality of Algerian sorghum cultivars. The starch fractions have acceptable nutritional value with good in vitro digestibility characteristics suitable for human health and nutrition.

## Introduction

Sorghum is a staple food crop for many of the world's poorest people in Africa and Asia. Many studies showed that it will become the alternative for wheat in the future (Beta et al. 2001c; Sang et al., 2008; Taylor et al. 2006). There are several indications that define grain quality, such as structural and biochemical characteristics, digestibility, bioavailability of nutrients, and antinutritional factor. The structure of sorghum kernels varies significantly because of environmental and genetic factors. Shape, size, proportion, and nature of the endosperm, germ, and pericarp, the presence and absence of subcoat, and the color of the pericarp are all genetically determined (Rooney and Murthy 1982).

Several works have been conducted to study the kinetics of starch digestion from different grains by α-amylase (Goni et al. 1997; Frei et al. 2003; Ezeogu et al. 2005). Uncooked and cooked sorghum grains have been reported to have a lower starch digestibility when compared to maize and other cereal grains, such as rice and wheat (Ezeogu et al. 2005). Lower digestibility of cooked sorghum starch has been shown to affect the higher loss of human energy (MacLean et al. 1981). For instance, (Wong et al. 2009) studied the starch in vitro digestibility in sorghum by comparing the level of reducing sugar per hour, they found 40–47 mg reducing sugar/h (without pepsin pretreatment) and 60–85 mg reducing sugar/h (with pepsin pretreatment) in sorghum meal. While (Englyst et al. 1999) found that 72% of total glucose in wheat flour were digested after 120 min, and (Frei et al. 2003) showed that in cooked rice, 70–80% of initial starch were completely digested. Several studies suggested that the endosperm protein is almost responsible for low starch digestibility of sorghum grain (Zhang and Hamaker 1998; Elkhalifa et al. 1999; Ezeogu et al. 2005). Moreover, in vitro digestibility of starch was affected by the endosperm texture, structure of starch, and nonstarch components (Tester and Morrison 1994; Sarikaya et al. 2000; Ezeogu et al. 2005; Benmoussa et al. 2006).

The glycemic index (GI) is an in vitro measurement based on glycemic response to carbohydrate-containing foods, and allows ranking of food on the basis of the rate of digestion and absorption of carbohydrates that they contain (Jenkins et al. 1981; Englyst et al. 1992). In vitro methods have also been used to classify foods based on their digestion characteristics similar to the in vivo situation, and to identify slow release of carbohydrate in foods (Jenkins et al. 1984; Schweizer et al.^1988^). Food materials with GI values more than 70%, between 56% and 69% and lower than 55% were classified as high, medium, and low GI foods, respectively (Brand-Miller et al. 2003).

Many sorghum cultivars are cultivated in the Sahara of Algeria; in Tidikelt (In Salah), a hyperarid region, known to have temperatures ranging from 7.8 to 45.2°C, a very low annual rainfall rate (16.9 mm), and irrigation is done with saline water. In addition, these environmental factors affect the starch properties in different sorghum genotypes (Matsuki et al. 2003; Boudries et al. 2009; Belhadi et al. 2012). Actually, sorghum production in these marginalized areas depend on traditional harvesting and processing. Most of the harvest is used as animal feed and rarely for human consumption. In the past, wide range of traditional food products was made from sorghum including kisra, porridge, and couscous.

One of the objectives of our laboratory was the study of the nutritional and healthy quality traits of sorghum grains and the isolation of starch and protein fractions and their characteristics that benefit for food and nonfood uses (Boudries et al. 2009; Mokrane et al. 2009, 2010; Hadbaoui et al. 2010; Belhadi et al. 2012). In a previous work, the protein nutritional quality in seven sorghum cultivars cultivated in the Sahara of Algeria was assessed. High percentages of protein, up to 16% db, have been found in the studied cultivars with favorable amino acid composition. The measure of the in vitro pepsin digestibility showed that some cultivars exhibit high digestibility, whereas other cultivars were characterized by their low digestibility (Mokrane et al. 2010).

The aim of the present study was to evaluate the digestibility of starch of starch in Algerian sorghum grain cultivars, by investigating starch in vitro digestion in sorghum grain flour, assessing the in vitro digestion kinetic and evaluating the effect of cultivar differences on the kinetic parameters.

## Materials and Methods

### Materials

Nine samples of local sorghum [*Sorghum bicolor (L.) Moench*] cultivars were harvested from different localities from Tidikelt region in Algerian Sahara; Taarankoukou, In salah, El Malah, Foggaret Ezzoua, and Djafou. The samples were characterized by various colors and different harvest years (2010, 2011, and 2012). The sorghum grains were cleaned, representative 100 g sorghum samples were prepared and stored in a fridge at 4°C until used. The crops were grounded to flour in IKA Labotechik A10 (IKA Labortechnik JANKE and KUNKEL, Staufen, Germany) sample mill. The obtained flours were manually sieved over a 500-*μ*m sieve. All the reagents were of analytical grade.

### Methods

#### Sorghum grain quality

Some physical properties of sorghum grains (grain color, test weight, and 100-seed weight) were determined by descriptors (IBPGR and ICRISAT 1993). Endosperm texture was defined as the proportion of corneous relative to floury endosperm in the grain, which was determined subjectively by viewing sectioned kernels using a stereomicroscope, and comparing them to sorghum standards (Taylor and Taylor 2008). The kernels were classified as corneous, intermediate, or floury (International Association for Cereal Science and Technology 2008). The grain dimensions were measured and calculated with the method described by (Jain and Bal 1997). The test of tannin was determined by the chlorox bleach test developed by (Waniska et al. 1992). The moisture content of the sorghum was determined in triplicate by using approved procedures method 44-15A (American Association of Cereal Chemists 2000).

#### Starch analysis

##### Total starch

Total starch (TS) was determined enzymatically according to the modified method of Goni et al. (1997). The weighed sorghum flour sample (50 ± 0.1 mg) was dispersed in 6 mL KOH solution (2 mol/L), shaken in a vortex, left for 1–2 h at room temperature until starch total dissolution. A volume of 4 mL of sodium acetate buffer solution (0.4 mol/L, pH 4.75) containing 0.833 *μ*L of amyloglucosidase from *Aspergillus niger* (300 U/mL, sigma, A-7095) were added, and the mixture was introduced in a water bath at 60°C for 45 min with occasional shaking. A volume of 1 mL of the obtained solution was diluted to 10 mL with distilled water to obtain a glucose concentration lower than 100 *μ*g/mL. Glucose concentrations were determined using glucose oxidase–peroxidase Kit (Biomaghreb, Ibn khaldoun-Ariana, Tunisia). The absorbance was measured at a wavelength of 500 nm and the concentration of starch was obtained by multiplying the concentration of glucose by 0.9.

##### Resistant starch and digestible starch

Resistant starch (RS) and digestible starch (DS) was likewise determined according to the modified method described by Goni et al. (1996). 100 mg of sorghum flour was first incubated in 10 mL of HCl–KCl buffer solution pH 1.5. Then, for protein removal, 0.1 mL of a solution containing 100 mg of pepsin from porcine gastric mucosa (P7000; Sigma-Aldrich, St. Louis, MO) in 10 mL HCl–KCl buffer solution, pH 1.5, were added to each sample. The mixture (sorghum flour and pepsine) was first incubated in a shaking water bath at 40°C for 60 min, and then cooled at room temperature. A volume of 9 mL of phosphate buffer, pH 6.9, was added afterwards. Then, remaining starch was hydrolyzed at 37°C for 16 h by adding 1 mL of an enzyme solution containing 40 mg of *α*-amylase type VI.B from porcine pancreas (A3172, Sigma-Aldrich). The sample was centrifuged (15 min, 3000*g*) and the supernatant discarded, 3 mL of distilled water was then added to the residue. Then, 3 mL of 4 mol/L KOH mix was added and left for 30 min at room temperature with constant shaking. Approximately 5 mL of HCl solution (2 mol/L) was added and 4 mL of sodium acetate buffer solution (0.4 mol/L, pH 4.75) containing 0.833 *μ*L of amyloglucosidase from *A. niger* (300 U/mL; Sigma, A-7095) were added. Afterwards, it was mixed well and left for 45 min in water bath at 60°C with constant shaking, and set to centrifuge (15 min, 3000*g*). The residue was washed at least once with 10 mL of distilled water, centrifuged again, and the supernatant was combined to obtain a glucose concentration lower than 100 *μ*g/mL, the solution was extended to 100 mL. Glucose concentration was determined using glucose oxidase–peroxidase (Biomaghreb) Kit. The absorbance was measured at a wavelength of 500 nm and glucose concentration was converted into RS content by multiplying it with the factor 0.9. The DS was calculated by the difference between TS and RS.

### In vitro starch digestion

The in vitro starch digestion was determined according to the modified method of Goni et al. (1997). Around 300 mg of sorghum flour was prepared in large tubes and 25 mL of phosphate buffer solution pH 6.9 were added. To start starch hydrolysis, 5 mL of *α*-amylase (2 × 10^−4^ mg/mL) type VI.B from porcine pancreas (A3172; Sigma-Aldrich) was added. The prepared mixture was incubated at 37°C for 3 h with constant shaking. Aliquots of 0.2 mL were taken every 30 min for 3 h. *α*-Amylase was inactivated immediately by placing the tubes in a boiling water bath for 5 min. Then, 0.6 mL of 0.4 mol/L sodium acetate buffer solution (pH 4.75), and 0.2 mL of an enzyme solution containing 0.833 *μ*L of amyloglucosidase from *A. niger* (300 U/mL, Sigma, A-7095) were added. In order to hydrolyze digested starch into glucose, sample was incubated at 60°C for 45 min. Finally, the volume was adjusted to 20 mL with distilled water and glucose concentration in the digesta was measured within the range (25–100 *μ*g/mL) using the oxidase–peroxidase Kit (Biomaghreb, Tunisia). Digested starch at time *t* (*D*_*t*_) (g/100 g dry starch) was calculated by equation (1).


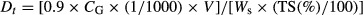
(1)

where *C*_G_**,** glucose concentration (*μ*g/mL); 0.9, represents stoichiometric constant of glucose content conversion into starch; 1/1000, the conversion from *μ*g to mg; *V*, volume of digesta (mL); *W*_s_, weight of sample (mg); TS (%), the total starch expressed as percentage in dry matter.

### Modeling of starch digestograms

The first-order exponential model in kinetic study has been used to estimate starch hydrolysis or glycemic indices in food and feed studies (Goni et al. 1997; Frei et al. 2003; Ezeogu et al. 2005), and more recently, (Mahasukhonthachat et al. 2010) included digested starch parameter *D*_t_ in this model.

However, this model can be modified (Eq. 2) to introduce digested starch *D*_*t*_ at time *t*.


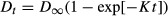
(2)

where *D*_**∞**_**,** digested starch at infinite time (g/100 g dry starch); *K*, constant rate (min^−1^); *t*, time (min).

*K* and *D*_∞_ were determined by LOS method “log of slope” described by (Butterworth et al. 2012). The differentiation of Eq. 2 gives Eq. 3:


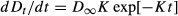
(3)

This first derivative represents the slope of a digestibility curve at time t, inserting logarithmic to Eq. (3) get Eq. (4):


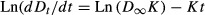
(4)

The glycemic and hydrolysis indices use the area under the hydrolysis curve (min g/100 g dry starch) (AUC_exp_), which is obtained by integrating Eq. (2) between times *t*_0_ = 0 min and *t*_f_ = 180 min getting Eq. (5):


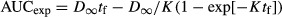
(5)

The hydrolysis index (HI), expressed as the ratio of the AUC_exp_ of the sample from 0 to 180 min to the area under the hydrolysis curve of white bread (∼15500 min g/100 g dry starch), was used in calculating GI using an equation (GI_HI_ = 39.51 + 0.570 HI) adapted from (Goni et al. 1997). Another equation (GI_H90_ = 39.21 + 0.803 *D*_90_) was also obtained from (Goni et al. 1997) to calculate GI_H90_ using a single-point measurement of digested starch (g/100 g dry starch) at 90 min; subscripts _th_ and _exp_ are, respectively, theoretical and experimental values. Consequently, an average GI_avg_, was defined as (GI_HI_ + GI_H90_)/2.

### Statistical analysis

All the parameters of sorghum grain quality and starch analysis were measured in three replicates and expressed as mean ± SD. The data analyses were performed with the SigmaPlot V.10.0 (Systat Software Inc, Chicago, Illinois, USA) for Windows.

## Results and Discussion

### Sorghum grain quality

Grain colors were white, red, and mixed (white and red), this characteristic is controlled genetically and modified by environmental conditions during and after maturation (Rooney and Miller 1982). As shown in Table 1, the weight of 100-kernel varied from 2.77 ± 0.1 to 3.44 ± 0.06 g with a mean value of 3.10 g. In International Crops Research Institute for Semi-Arid-Tropics (ICRISAT) laboratory, 100 sorghum samples were analyzed by Jambunathan et al. 1981 a wide range in 100-kernel weight was obtained 1.3–5.7 g with a mean value of 2.8 g (Achaya 1984). The results in the nine studied Algerian cultivars were generally higher than the mean value (Table 1). Mean test weight in our study ranged from 700.05 ± 2.19 to 733.78 ± 3.01 g/L, (Table 1).The differences in test weight value is probably due to growing conditions and genetics, as suggested by (Buffo et al. 1998).

**Table 1:** Harvest date, locality, grain color, test weight, 100-kernel weight, and endosperm texture of nine sorghum cultivars

							Endosperm texture (%)		
							
No.	Sorghum cultivars	Harvest date	Locality	Grain color	Test weight (g/L)	100-Kernel weight (g)	Corneous	Intermediate	Starchy
1	SB10TA	2010	Taarankoukou	White	700.05 ± 2.19	3.23 ± 0.17	10	85	5
2	SB10AS	2010	In Salah	White	707.90 ± 3.75	3.25 ± 0.06	80	20	0
3	SR10AS	2010	In Salah	Red	722.58 ± 3.13	2.77 ± 0.10	25	70	5
4	SM10AS	2010	In Salah	Red and White	718.16 ± 7.16	3.44 ± 0.06	5	75	20
5	SB11TA	2011	Taarankoukou	White	708.19 ± 2.62	3.07 ± 0.10	55	35	10
6	SB11MA	2011	El Malah	White	721.34 ± 2.20	2.82 ± 0.09	0	80	20
7	SB12FE	2012	Foggarat Ezzoua	White	733.78 ± 3.01	3.44 ± 0.04	75	25	0
8	SR12DJ	2012	Djafou	Red	727.56 ± 8.32	2.87 ± 0.14	0	80	20
9	SR12AS	2012	In Salah	Red	727.63 ± 9.61	3.06 ± 0.04	45	55	0

Table 2 shows the mean values of the three principal dimensions of specific grades of sorghum grain samples. The average values obtained for the major, minor, and medium diameters were 4.70, 2.50, and 3.97 mm, respectively. This means that the value is within the range reported by (Mwithiga and Sifuna 2006). The range of volume and surface area in the sorghum grain samples, varied, respectively, from 17.36 ± 5.57 to 21.91 ± 3.73 mm^3^ and 33.26 ± 6.67 to 38.99 ± 4.35 mm^2^ as shown in Table 2. Our results were lower than the mean values of volume and surface area 29.62 ± 4.37 mm^3^ and 74 ± 8 mm^2^ reported by (Ndiriki and Mohammed 2005) for sorghum cultivated in Nigeria.

**Table 2:** Shape expressed as *L* (major diameter, mm), *T* (minor diameter, mm), *W* (medium diameter, mm)

No.	Sorghum cultivars	Harvest date	Locality	*L* (mm)	*T* (mm)	*W* (mm)	*V* (mm^3^)	*S* (mm^2^)
1	SB10TA	2010	Taarankoukou	4.51 ± 0.34	2.43 ± 0.29	3.86 ± 0.47	17.36 ± 5.57	33.26 ± 6.67
2	SB10AS	2010	In Salah	4.42 ± 0.29	2.55 ± 0.25	3.91 ± 0.48	18.41 ± 5.07	34.41 ± 5.99
3	SR10AS	2010	In Salah	4.82 ± 0.42	2.39 ± 0.19	3.79 ± 0.32	16.90 ± 3.70	33.34 ± 4.79
4	SM10AS	2010	In Salah	4.82 ± 0.27	2.60 ± 0.22	4.31 ± 0.25	21.91 ± 3.73	38.99 ± 4.35
5	SB11TA	2011	Taarankoukou	4.48 ± 0.35	2.57 ± 0.28	3.89 ± 0.50	18.34 ± 4.31	34.52 ± 4.90
6	SB11MA	2011	El Malah	4.57 ± 0.65	2.57 ± 0.29	3.97 ± 0.27	18.75 ± 4.60	34.94 ± 7.00
7	SB12FE	2012	Foggarat Ezzoua	4.89 ± 0.32	2.50 ± 0.32	4.23 ± 0.23	20.56 ± 4.43	37.61 ± 4.81
8	SR12DJ	2012	Djafou	4.91 ± 0.38	2.46 ± 0.37	4.03 ± 0.30	19.01 ± 4.92	35.87 ± 5.52
9	SR12AS	2012	In Salah	4.89 ± 0.37	2.45 ± 0.21	3.78 ± 0.34	17.53 ± 3.66	34.19 ± 4.66

The moisture content in sorghum grains ranged from 08.32% to 10.17% (Table 3). Visual examination of endosperm texture varied in percentage of corneous (0–80%), intermediate (20–85%), and starchy (0–20%) fractions (Table 1). This variation in endosperm texture indicate that the grains should be classified as mixed and intermediate endosperm type as described by International Association for Cereal Science and Technology (2008). The test of tannin showed that the nine sampled sorghum grains were free from tannins. According to Federal Grain Inspection Service (FGIS/GIPSA), the grains were classified “white sorghums” without pigmented testa. Thus, the sampled sorghum grains might be more suitable for milling and unleavened breads to produce tortillas (Rooney and Miller 1982; Gomez et al. 1997).

**Table 3:** Moisture, total starch (TS), resistant starch (RS), digestible starch (DS), and starch digestibility (%) in sorghum flours

No.	Sorghum cultivars	Moisture (%)	TS (%)	RS (%)	DS (%)	Starch digestibility (%)
1	SB10TA	08.80	70.28 ± 3.02	4.91 ± 0.34	65.37	93.01
2	SB10AS	08.69	67.79 ± 3.01	3.42 ± 0.10	64.37	94.95
3	SR10AS	08.32	74.42 ± 3.65	5.28 ± 0.22	69.14	92.90
4	SM10AS	08.67	70.11 ± 3.69	3.62 ± 0.04	66.49	94.83
5	SB11TA	09.60	67.67 ± 1.25	2.55 ± 0.15	65.12	96.22
6	SB11MA	09.91	74.33 ± 4.82	4.63 ± 0.13	69.70	93.76
7	SB12FE	08.41	74.12 ± 1.54	4.77 ± 0.37	69.35	93.56
8	SR12DJ	10.17	72.49 ± 2.37	4.81 ± 0.23	67.68	93.36
9	SR12AS	09.42	74.82 ± 0.75	7.98 ± 0.44	66.82	89.30

Large variation for grain qualitative traits was observed in Algerian sorghum cultivars. Based on these variations probably due to environmental conditions, high genotype diversity is found among the cultivars (Rooney and Miller 1982).

### Starch analysis and measurement of nutritionally important starch fractions

Total starch (TS) content of sorghum flour ranged from 67.67% to 74.82% db with a mean value of 71.78% (Table 3). The grain chemical composition of sorghum genotypes from the world collection at ICRISAT showed that starch composition was between 55.6% and 75.2% with a mean value of 69.5% (FAO 1995). Dicko et al. (2006) evaluated the total starch content in 50 varieties of sorghum, and obtained a mean of 63% with a range of 57–69%. When compared to our results, the total starch content in the Algerian sorghum samples was higher than the mean value. Moreover, the grain starch contents in the nine studied sorghum cultivars were higher than those observed in wheat (65%), rye (60%), and barley (55%) (Choct and Hughes 2000). However, our samples exhibited lower total starch content than maize (75%) and rice (80%) (Choct and Hughes 2000).

Some nutritionally important starch fractions in the grains are shown in Table 3. DS ranged between 64.37% and 69.70% with a mean value of 67.11%. The DS contents in the nine studied cultivars were higher than those observed in beans (26.18%) and boiled potatoes (56.76%), but lower than rice (79.69%) and spaghetti (71.08%) (Goni et al. 1997). The digestibility percentage (%) in samples ranged from 89.30% to 96.22%, with a mean value of 93.54%, the digestibility is similar to sorghum grown in Australia from 2004 and 2005 harvest (Bryden et al. 2009), and much higher than the average of 11 sorghum samples, grown in the southwestern part of the United States with the value of 16.2% (Osman et al. 1970). In a previous work, the samples from the same region In Salah (Tidikelt) showed the highest protein in vitro digestibility and then the highest amino acid score (AAS) (Mokrane et al. 2010). The relatively high starch and protein digestibility and high AAS suggest that this sorghum would be useful in foods such as for weaned infants.

The resistant starch value (RS) obtained in samples: SB10AS, SM10AS, SB11TA, SB10TA, SB11MA, SB12FE, SR12DJ was found to be from 2.5% to 5%, which is considered intermediate, while it ranged from 5% to 15% in SR10AS and SR12AS which is considered high, according to the classification of resistant starch content as suggested by (Goni et al. 1996). Several factors can explain the difference found in the resistant starch quantities, as follows: interaction of starch with different components present in the food system such as proteins, fats, botanical source of starch, and storage conditions (Sajilata et al. 2006; Perera et al. 2010). Two sorghum samples contained elevated RS (5–15%) in grain sorghum flour. This resistance is desired in other applications to health problems for diabetics and prediabetic subjects and to fight human obesity (Jenkins et al. 2008; Hendrich 2010).

In this study, sorghum grains have a high starch content, variability in DS and RS displayed among cultivars. Thus, all the accessions are genotype and quality traits effects on nutritionally important starch fractions.

### In vitro kinetic starch digestion and Modeling

**Figure 1:**
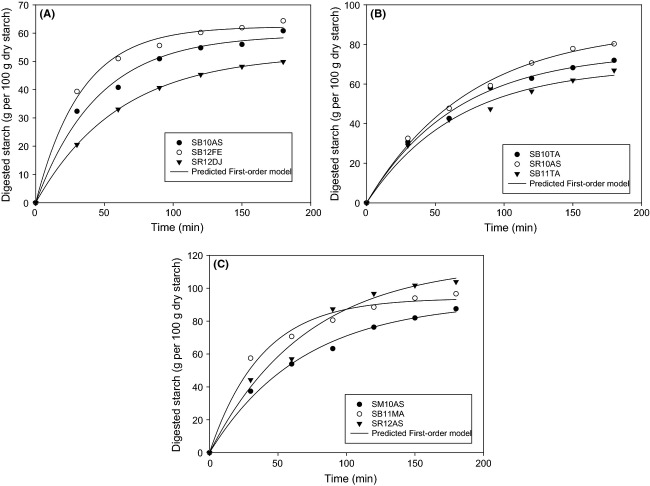
Hydrolysis curves of the samples showing the differences in maximum starch digested *D*_t_ (g/100 g dry starch) of the nine sorghum cultivars and predictability of the first-order model. (A) 0 < *D*_*t*_ < 60, (B) 60 < *D*_t_ < 80, and (C) 80 < *D*_t_ < 100.

The starch hydrolysis curves for grain samples are shown in (Fig. 1). The reactions undergo a first-order model, a considerable amount of starch digested within the duration of the substrate–enzyme contact. (Fig. 1). First-order model properties were demonstrated in in vitro starch digestion of raw and processed food and feed (Goni et al. 1997; Frei et al. 2003; Ezeogu et al. 2005). According to digested starch values after 180 min, the sorghum samples were classified in (Fig. 1) into three types: digestibility more than 80% (*D*_180_ > 80) (SM10AS, SB11MA, SR12AS), digestibility between 60% and 80% (*D*_180_ 60–80) (SB10TA, SB11TA, SR10AS), and digestibility lower than 60% (*D*_180_ < 60) (SB10AS, SR12DJ, SB12FE).

Hence, the modeling of starch digestion kinetic is required to derive more quantitative information on digestibility properties. The first-order kinetic model was suitable, including the estimated parameters *D*_∞_, *K*, the hydrolysis index HI, and GI_avg_. The digested starch at infinite time *D*_∞_ varied from 52.58 to 102.13 (g/100 g dry starch), K ranged between 0.011 and 0.0245 min^−1^, and the hydrolysis index (HI) ranged from 41.55% to 76.93% (Table 4).

**Table 4:** Digestibility and glycemic parameters of the first-order kinetic model for the sorghum cultivars (g/100 g dry starch)

No.	Sorghum cultivars	*K* (min^−1^)	*D*_∞_	AUC	HI (%)	*D*_90 exp_	*D*_90th_	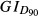	GI_HI_	GI_avg_
1	SB10TA	0.0131	69.11	7663.35	49.44	58.21	47.85	85.95	67.69	76.86
2	SB10AS	0.0245	56.88	7944.99	51.25	50.92	50.60	80.10	68.72	74.41
3	SR10AS	0.0146	88.66	10342.78	66.61	59.16	64.83	86.71	77.47	82.09
4	SM10AS	0.0123	102.13	10987.38	70.88	63.23	68.37	89.98	79.91	84.94
5	SB11TA	0.0110	83.71	8508.50	54.89	47.37	52.60	77.25	70.79	74.02
6	SB11MA	0.0177	94.76	11924.43	76.93	80.55	75.49	103.89	83.36	93.62
7	SB12FE	0.0240	58.80	8166.58	52.68	55.60	52.01	83.85	69.53	76.69
8	SR12DJ	0.0165	52.58	6441.21	41.55	36.78	40.67	68.74	63.19	65.97
9	SR12AS	0.0173	86.03	10733.47	69.25	87.29	67.89	109.30	78.98	94.14

The kinetic constant *K* of amylolysis has been proposed as a reliable index of the inherent susceptibility of flour starches to amylase hydrolysis (Goni et al. 1997; Frei et al. 2003). A wide variation was observed in the nine sorghum flours in amylase susceptibility, from the lowest in SR12DJ (41.55%) to the highest in SB11MA (76.93%). HI is lowest in SB10AS and SB12FE was probably due to vitreous (corneous) endosperm, which reduces *α*-amylase access to the starch and the formation of more complex and restrictive prolamin protein network (Ezeogu et al. 2005). These variations in the digestibility of the sorghum flours are due to the grain quality differences in the sampled sorghum cultivars. (Rooney and Pflugfelder 1986).

According to the classification of GI content as suggested by (Brand-Miller et al. 2003), the GI in eight samples highly ranged from 74.02 to 94.14, while it was medium in one sample SR12DJ (65.97) (Table 4). In the 2002 edition of the international table of GI and Glycemic Load as reported by (Foster-Powell et al. 2002), the GI in roasted jowar bread made from jowar flour (*Sorghum vulgare*) was found to be 110, while in maize (*Zea mays*) was about 85, in flour made into chapatti (India) was 60 ± 8 and 94 for brown rice (Canada). (Mani et al. 1993) also found that GI ranged from 55 ± 13 to 104 ± 13 after testing six commonly consumed sorghum foods of India. The results indicate that sorghum samples in our work are classified as having a high GI.

The HI and GI_avg_ values of sorghum flours grown in Algeria were much higher, possibly due to differences in genetic source, growing conditions, and the employed methods to determine HI and GI_avg_. This result suggests that there are good opportunities for utilization of sorghum grain, grown in Sahara of Algeria, for human health and nutrition purposes.

## Conclusions

This study pointed out that the differences in structural, physical, and biochemical properties of sorghum grains demonstrated the diversity of genotype in local sorghum cultivars in Algeria. Moreover, the measure of in vitro starch digestibility showed that the nine local cultivars exhibited high digestibility up to 90%. First-order model can assess the modeling starch digestion of uncooked sorghum flours. Nutritionally important starch fractions and starch digestion can be affected by many factors including genotype, environmental conditions, structure of starch, and nonstarch components. Finally, the digestibility properties of the Algerian sorghum cultivars showed a high diversity and might have implications for human health and nutrition.

## References

[b1] Achaya KT (1984). http://archive.unu.edu.

[b2] American Association of Cereal Chemists (2000).

[b3] Belhadi B, Djabali D, Souilah R, Yousfi M, Nadjemi B (2012). Three small-scale laboratory steeping and wet-milling procedures for isolation of starch from sorghum grains cultivated in Sahara of Algeria. Food Bioprod. Process.

[b4] Benmoussa M, Suhendra B, Aboubacar A, Hamaker BR (2006). Distinctive sorghum starch granule morphologies appear to improve raw starch digestibility. Starch/Stärke.

[b5] Beta T, Obilana AB, Corke H (2001c). Genetic diversity in properties of starch from Zimbabwean sorghum landraces. Cereal Sci.

[b6] Boudries N, Belhaneche N, Nadjemi B, Deroanne C, Mathlouthi M, Roger B (2009). Physicochemical and functional properties of starches from sorghum cultivated in the Sahara of Algeria. Carbohydr. Polym.

[b7] Brand-Miller J, Wolever TMS, Foster-Powell K, Colagiuri S (2003). The new glucose revolution.

[b8] Bryden WL, Selle PH, Cadogan DJ, Li X, Muller ND, Jordon DR (2009).

[b9] Buffo RA, Weller CL, Parkhurst AM (1998). Relationships among grain sorghum quality factors. Cereal Chem.

[b10] Butterworth PI, Warren FJ, Grassby T, Patel H, Ellis RP (2012). Analysis of starch amylolysis using plots for first-order kinetics. Carbohydr. Polym.

[b11] Choct M, Hughes B (2000).

[b12] Dicko MH, Gruppen H, Traore AS, Voragen AGJ, Van Berkel WJH (2006). Sorghum grain as human food in Africa: relevance of content of starch and amylase activities. Afr. J. Biotechnol.

[b13] Elkhalifa AO, Chandrashekar A, Mohamed BE, El Tinay AH (1999). Effect of reducing agents on the in vitro protein and starch digestibilities of cooked sorghum. Food Chem.

[b14] Englyst HN, Kingman SM, Cummings JH (1992). Classification and measurement of nutritionally important starch fractions. Eur. J. Clin. Nutr.

[b15] Englyst KN, Englyst HN, Hudson GJ, Cole TJ, Cummings JH (1999). Rapidly available glucose in foods: an in vitro measurement that reflects the glycemic response. Am. J. Clin. Nutr.

[b16] Ezeogu LI, Duodua KG, Taylor JRN (2005). Effects of endosperm texture and cooking conditions on the in vitro starch digestibility of sorghum and maize flours. J. Cereal Sci.

[b17] FAO (1995). Sorghum and millets in human nutrition, collection FAO.

[b18] Foster-Powell K, Holt SHA, Brand-Mille CJ (2002). International table of glycemic index and glycemic load values: 2002. Am. J. Clin. Nutr.

[b19] Frei M, Siddhuraju P, Becker K (2003). Studies on the in vitro starch digestibility and glycemic index of six different indigenous rice cultivars from the Philippines. Food Chem.

[b20] Gomez MI, Obilana AB, Martin DF, Madzvamuse M, Monyo ES (1997). Manual of laboratory procedures for quality evaluation of sorghum and pearl millet.

[b21] Goni I, Garcia-Diz L, Manas E, Saura-Calixto F (1996). Analysis of resistant starch a method for foods and food products. Food Chem.

[b22] Goni I, Garcia-Alonsa A, Saura-Calixto F (1997). A starch hydrolysis procedure to estimate glycemic index. Nutr. Res.

[b23] Hadbaoui Z, Djeridane A, Yousfi M, Saidi M, Nadjemi B (2010). Fatty acid, tocopherol composition and the antioxidant activity of the lipid extract from the sorghum grains growing in Algeria. Mediterr. J. Nutr. Metab.

[b24] Hendrich S (2010). Battling obesity with resistant starch. Food Technol.

[b25] IBPGR, and ICRISAT (1993). Descriptors for sorghum [Sorghum bicolor (L.) Moench].

[b26] ICC, International Association for Cereal Science and Technology (2008).

[b27] Jain RK, Bal S (1997). Properties of pearl millet. J. Agric. Eng. Res.

[b54] Jambunathan R, Singh U, Subramanian V (1981). Grain quality of sorghum, Pearl Millet, Pigean-Pea, and chick-Pea. Proceedings of a workshop on interfaces between agriculture, Nutrition, and Food science.

[b28] Jenkins DJA, Wolever TMS, Taylor RH, Barker HM, Fielden H, Baldwin JH (1981). Glycemic index of foods: a physiological basis for carbohydrate exchange. Am. J. Clin. Nutr.

[b29] Jenkins DJA, Wolever TMS, Thorne MJ, Jenkins AL, Wong GS, Josse RG (1984). The relationship between glycemic response, digestibility and factors influencing the dietary habits of diabetics. Am. J. Clin. Nutr.

[b30] Jenkins DJA, Kendall CWC, McKeown-Eyssen G, Josse GR, Silverberg GJ, Booth GL (2008). Effect of a low-glycemic index or a high-cereal fiber diet on type 2 diabetes: a randomized trial. J. Am. Med. Assoc.

[b31] MacLean WC, de Romana GL, Placko RP, Graham GG (1981). Protein quality and digestibility of sorghum in preschool children: balance studies and plasma free amino acids. J. Nutr.

[b32] Mahasukhonthachat K, Sopade PA, Gidley MJ (2010). Kinetic of starch digestion in sorghum as affected by particle size. J. Food Eng.

[b33] Mani UV, Ficn BM, Damle SS, Mani I (1993). Glycaemic index of some commonly consumed foods in Western India. Asia Pac. J. Clin. Nutr.

[b34] Matsuki J, Yasui T, Kohyama K, Sasaki T (2003). Effects of environmental temperature on structure and gelatinisation properties of wheat starch. Cereal Chem.

[b35] Mokrane H, Lagrain B, Gebruers K, Courtin CM, Brijs K, Proost P (2009). Characterization of kafirins in Algerian sorghum cultivars. Cereal Chem.

[b36] Mokrane H, Amoura H, Belhaneche-Bensemra N, Courtin CM, Delcour JA, Nadjemi B (2010). Assessment of Algerian sorghum protein quality [Sorghum *bicolor (L.) Moench*] using amino acid analysis and in vitro pepsin digestibility. Food Chem.

[b37] Mwithiga G, Sifuna MM (2006). Effect of moisture content on the physical properties of three varieties of sorghum seeds. J. Food Eng.

[b38] Ndiriki VIO, Mohammed SS (2005). Determination of selected physical properties and their relationship with moisture content for sorghum crop. J. Food Technol.

[b39] Osman HF, Theurer B, Hale WH, Mehen SM (1970). Influence of grain processing on in vitro enzymatic starch digestion of barley and sorghum grain. J. Nutr.

[b40] Perera A, Meda V, Tyler RT (2010). Resistant starch: a review of analytical protocols for determining resistant starch and of factors affecting the resistant starch content of foods. Food Res. Int.

[b41] Rooney LW, Miller FR (1982).

[b42] Rooney LW, Murthy DS (1982).

[b43] Rooney LW, Pflugfelder RL (1986). Factors affecting starch digestibility with special emphasis on sorghum and corn. J. Anim. Sci.

[b44] Sajilata MG, Singhal RS, Kulkami PR (2006). Resistant starch. A review. Compr. Rev. Food Sci. Food Saf.

[b53] Sang Y, Bean S, Seib PA, Pederson J, Shi YC (2008). Structure and functional properties of sorghum starches differing in amylose content. J. Agric. Food Chem.

[b45] Sarikaya E, Higasa T, Adachi M, Mikami B (2000). Comparison of degradation abilities of *α*- and *β*-amylases on raw starch granules. Process Biochem.

[b46] Schweizer TF, Reimann S, Wursch P (1988). Definition and measurement of a starch digestion index and a study of factors determining starch digestion rates in foods. Food Sci. Technol.

[b47] Taylor JRN, Taylor J (2008). http://www.intsormil.org.

[b48] Taylor JRN, Schober TJ, Bean SR (2006). Novel food and non-food uses for sorghum and millets. J. Cereal Sci.

[b49] Tester RF, Morrison WR (1994). Properties of damaged starch granules. V. composition and swelling of fractions of wheat in water at various temperatures. J. Cereal Sci.

[b50] Waniska RD, Hugo LF, Rooney LW (1992). Practical methods to determine presence of tannins in sorghum. J. Appl. Poult. Res.

[b51] Wong JH, Lau T, Cai N, Singh J, Pedersen JF, Vensel WH (2009). Digestibility of protein and starch from sorghum (Sorghum bicolor) is linked to biochemical and structural features of grain endosperm. J. Cereal Sci.

[b52] Zhang G, Hamaker BR (1998). Low *α*-amylase starch digestibility of cooked sorghum flours and the effect of protein. Cereal Chem.

